# Multimodality imaging of the right ventricular outflow tract haemangioma requiring pulmonary valve replacement

**DOI:** 10.1093/ehjcr/ytag123

**Published:** 2026-02-14

**Authors:** Rui Katano, Satonori Tsuneta, Satoru Wakasa, Atsushi Tada

**Affiliations:** Department of Cardiovascular Medicine, Faculty of Medicine and Graduate School of Medicine, Hokkaido University, Kita 15, Nishi 7, Kita-ku, Sapporo, Hokkaido 060-8638, Japan; Department of Radiology, Graduate School of Dental Medicine, Hokkaido University, Kita 13, Nishi 7, Kita-ku, Sapporo, Hokkaido 060-8586, Japan; Department of Cardiovascular Surgery, Faculty of Medicine and Graduate School of Medicine, Hokkaido University, Kita-15, Nishi-7, Kita-ku, Sapporo, Hokkaido 0608638, Japan; Department of Cardiovascular Medicine, Faculty of Medicine and Graduate School of Medicine, Hokkaido University, Kita 15, Nishi 7, Kita-ku, Sapporo, Hokkaido 060-8638, Japan

## Case description

**Figure 1:**
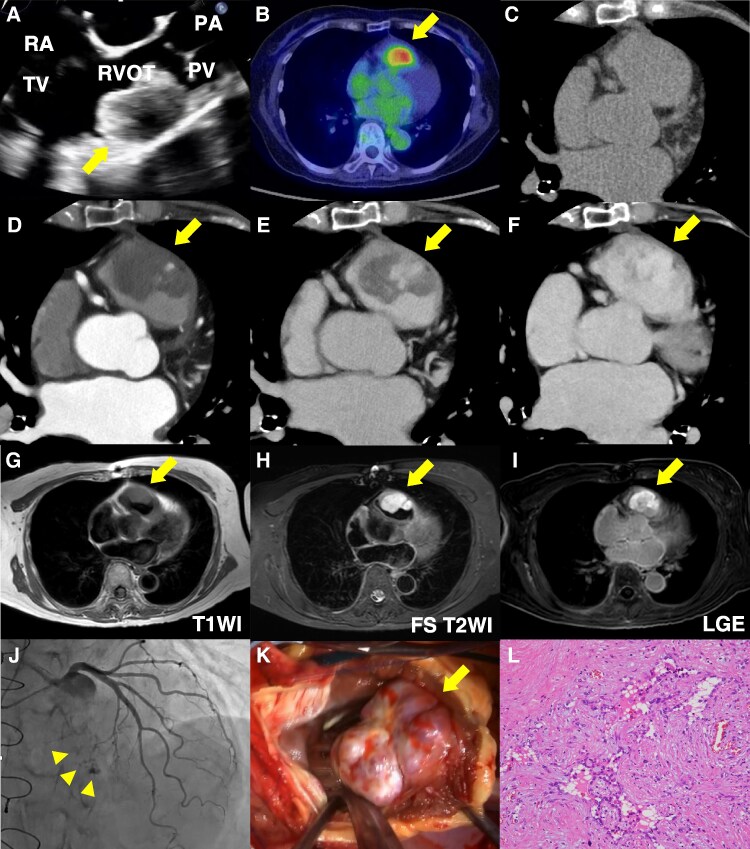
Multimodality imaging characterizes a right ventricular outflow tract tumour and suggests a benign aetiology pre-operatively (*A–J*). Transoesophageal echocardiography (*A*), 18F-FDG PET (*B*), computed tomography: non-contrast computed tomography (*C*), arterial phase (*D*), equilibrium phase (*E*), and delayed phase (*F*) of contrast-enhanced computed tomography, cardiac MRI: T1-weighted image (*G*), fat-suppressed T2-weighted image (*H*), late gadolinium enhancement (*I*), and coronary angiography (*J*) demonstrate a broad-based mass arising from the right ventricular outflow tract. Non-contrast computed tomography showed slightly low attenuation (38 HU), which was lower than that of the blood pool (46 HU) (*C*). Intra-operative photograph shows the mass adjacent to the pulmonary valve anterior leaflet annulus (*K*). Post-operative histopathology reveals proliferation of vascular channels, forming cavernous, slit-like, and papillary spaces, without significant cytologic or architectural atypia (*L*). RVOT, right ventricular outflow tract; HU, Hounsfield units; PV, pulmonary valve; TV, tricuspid valve; PA, pulmonary artery; RA, right atrium; T1WI, T1-weighted image; FS-T2WI, fat-suppressed T2-weighted image; LGE, late gadolinium enhancement.

A 76-year-old woman was referred for evaluation of a suspected right ventricular tumour identified on computed tomography (CT). She was asymptomatic, and tumour markers were negative. Transoesophageal echocardiography showed a broad-based, 3-cm mass in the right ventricular outflow tract (RVOT), located immediately beneath the pulmonary valve, without RVOT obstruction (*Figure 1A*; Supplementary material online, *Video S1*). ^18^F-FDG PET demonstrated localized uptake (SUVmax 3.73; *Figure 1B*). Non-contrast CT showed slightly low attenuation (38 Hounsfield units) (*Figure 1C*), and contrast-enhanced CT demonstrated nodular peripheral enhancement in the arterial phase, followed by progressive centripetal fill-in in the later phases (*Figure 1D–F*) and no evidence of pulmonary embolism. Cardiac MRI demonstrated low T1 signal (*Figure 1G*) and high fat-suppressed T2 signal (*Figure 1H*), with uniform delayed enhancement (*Figure 1I*), which, in the absence of predisposing factors, helped exclude thrombus. Coronary angiography identified feeding vessels arising from a septal branch (*Figure 1J*; Supplementary material online, *Video S2*). Pre-operative catheter-based biopsy was not performed due to risks of tumour-related bleeding or systemic embolization.

Given the working diagnosis of a primary cardiac tumour, surgical resection was performed (*Figure 1K*). Owing to its proximity to the pulmonary valve annulus, en bloc excision with bioprosthetic valve replacement and RVOT reconstruction was necessary to avoid incomplete resection or significant regurgitation. Post-operative histopathology revealed proliferation of vascular channels, forming cavernous, slit-like, and papillary spaces, without significant cytologic or architectural atypia (*Figure 1L*). Immunostaining was positive for CD31, CD34, and ERG, but negative for AE1/AE3, EMA, and D2-40. With low Ki-67 labelling index (approximately 2%) and wild-type p53 pattern, these findings confirmed the diagnosis of haemangioma.

Pre-operative distinction between benign and malignant cardiac tumours remains challenging, particularly for right-sided masses. This case demonstrates that multimodality imaging, integrating morphological features, enhancement patterns, metabolic activity, and malignancy prediction scores,^1^ can support a benign diagnosis and inform surgical planning.

## Supplementary Material

ytag123_Supplementary_Data

## Data Availability

No new data were generated or analysed in support of this research.
