# Supplementary figures and images for: Multimodality imaging of the right ventricular outflow tract haemangioma requiring pulmonary valve replacement
Source: Eur Heart J Case Rep. 2026 Feb 14;10(3):ytag123. doi: 10.1093/ehjcr/ytag123 (PMC12970997; doi:10.1093/ehjcr/ytag123)

## Slide 1
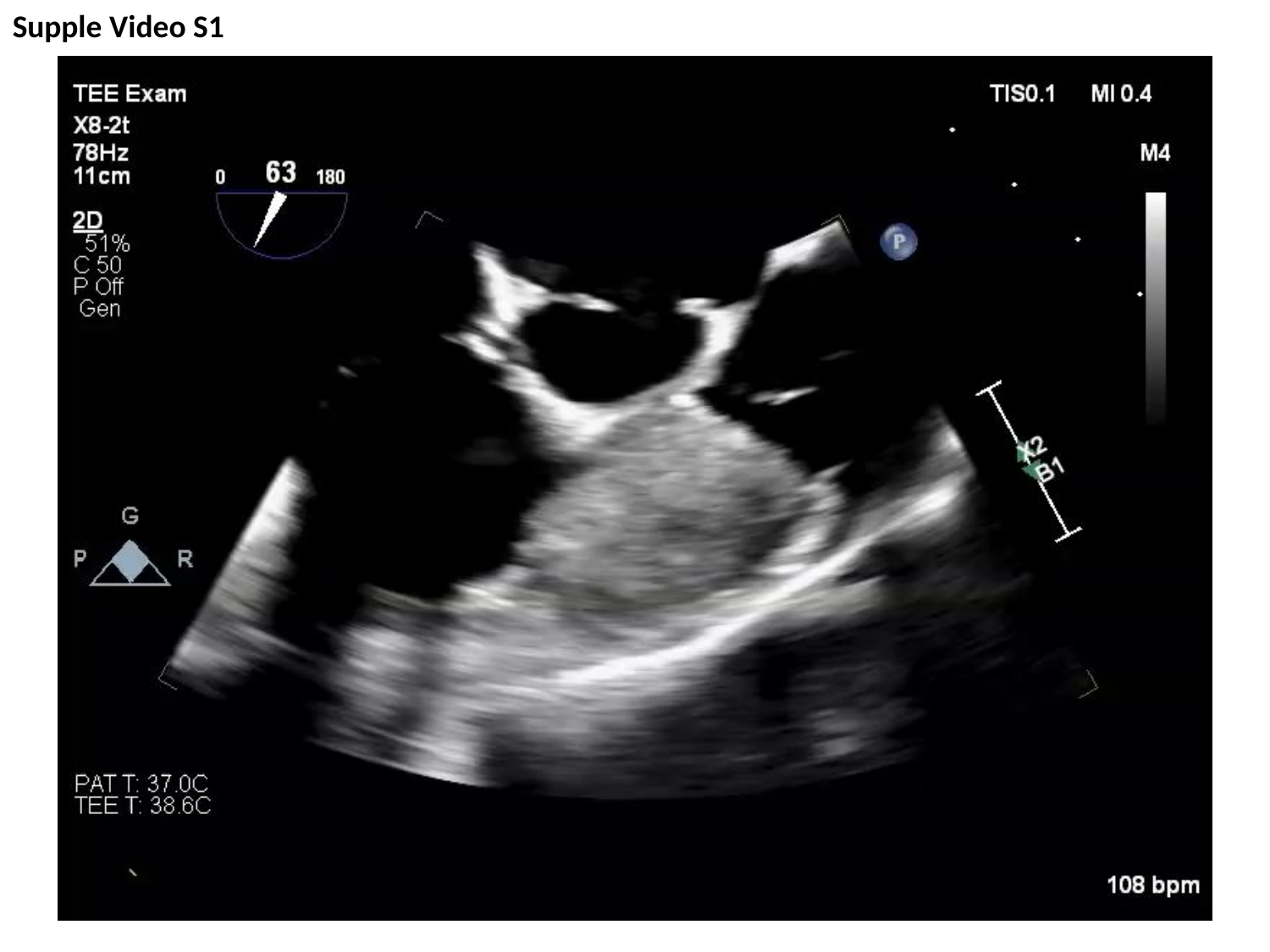

Supple Video S1

## Slide 2
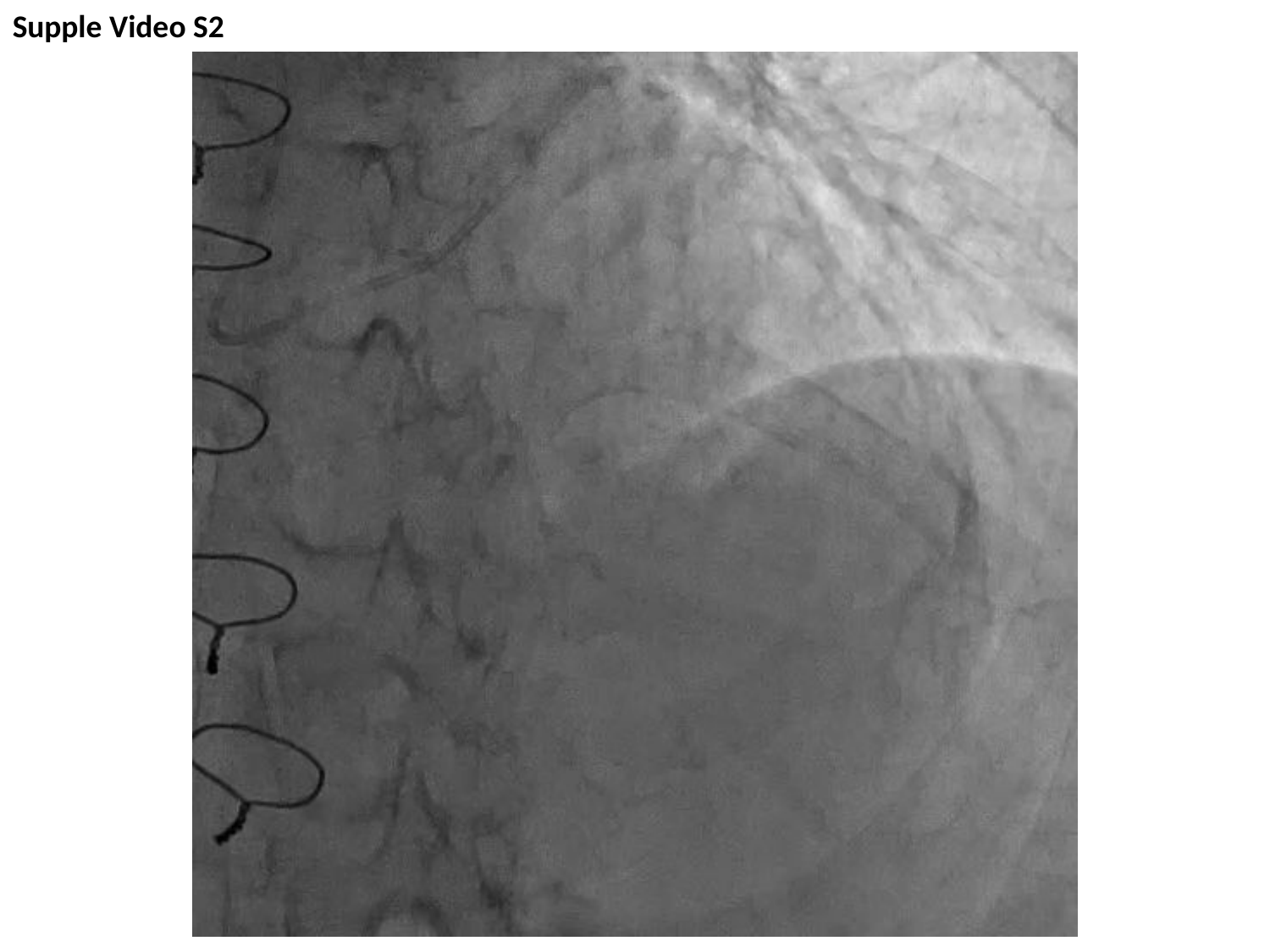

Supple Video S2

Supplement: ytag123_Supplementary_Data [file ytag123_supplementary_data.pptx]
